# On-Line Solid Phase Extraction High Performance Liquid Chromatography Method Coupled With Tandem Mass Spectrometry for the Therapeutic Monitoring of Cannabidiol and 7-Hydroxy-cannabidiol in Human Serum and Saliva

**DOI:** 10.3389/fphar.2022.915004

**Published:** 2022-06-22

**Authors:** Valentina Franco, Michela Palmisani, Roberto Marchiselli, Francesca Crema, Cinzia Fattore, Valentina De Giorgis, Costanza Varesio, Paola Rota, Vincenza Flora Dibari, Emilio Perucca

**Affiliations:** ^1^ Department of Internal Medicine and Therapeutics, Clinical and Experimental Pharmacology Unit, University of Pavia, Pavia, Italy; ^2^ IRCCS Mondino Foundation, Pavia, Italy; ^3^ Department of Brain and Behavioral Sciences, University of Pavia, Pavia, Italy; ^4^ Department of Biomedical, Surgical and Dental Sciences, University of Milan, Milan, Italy; ^5^ B.S.N. Srl R&D Laboratory, Castelleone, Italy; ^6^ Department of Medicine, Austin Health, The University of Melbourne, Melbourne, VIC, Australia; ^7^ Department of Neuroscience, Monash University, Melbourne, VIC, Australia

**Keywords:** cannabidiol, 7-hydroxy-cannabidiol, antiseizure medications, HPLC-MS/MS, saliva, serum, on-line solid phase extraction

## Abstract

Cannabidiol is a novel antiseizure medication approved in Europe and the US for the treatment of seizures associated with Lennox-Gastaut syndrome, Dravet syndrome and tuberous sclerosis complex. We describe in this article a new and simple liquid chromatography-mass spectrometry method (LC-MS/MS) for the determination of cannabidiol and its active metabolite 7-hydroxy-cannabidiol in microvolumes of serum and saliva (50 μl), to be used as a tool for therapeutic drug monitoring (TDM) and pharmacokinetic studies. After on-line solid phase extraction cannabidiol, 7-hydroxy-cannabidiol and the internal standard cannabidiol-*d3* are separated on a monolithic C18 column under gradient conditions. Calibration curves are linear within the validated concentration range (10–1,000 ng/ml for cannabidiol and 5–500 ng/ml for 7-hydroxy-cannabidiol). The method is accurate (intraday and interday accuracy within 94–112% for cannabidiol, 91–109% for 7-hydroxy-cannabidiol), precise (intraday and interday precision <11.6% for cannabidiol and <11.7% for 7- hydroxy-cannabidiol) and sensitive, with a LOQ of 2.5 ng/ml for cannabidiol and 5 ng/ml for 7-hydroxy-cannabidiol. The stability of the analytes was confirmed under different storage conditions. Extraction recoveries were in the range of 81–129% for cannabidiol and 100–113% for 7-hydroxy-cannabidiol. The applicability of the method to TDM was demonstrated by analysis of human serum and saliva samples obtained from patients with epilepsy treated with cannabidiol.

## Introduction

Cannabidiol (CBD) is a new antiseizure medication (ASM) recently approved in Europe and the US for the treatment of seizures associated with Lennox-Gastaut syndrome, Dravet syndrome and tuberous sclerosis complex (Epidyolex, 2019; Epidiolex, 2020). The mechanisms by which CBD exerts antiseizure effects are unclear. CBD shows low affinity for the cannabinoid CB1 and CB2 receptors at therapeutic concentrations, and its antiseizure effects may be related to other actions such as inhibition of adenosine reuptake, G protein–coupled receptor 55 (GPR55) antagonism and desensitization of vanilloid type 1 (TRPV1) channels (Perucca, 2017; Franco and Perucca, 2019; Franco et al., 2021). CBD bioavailability is low and very variable (around 6% on average) due to extensive first-pass metabolism (Bialer et al., 2018), and increases 4-fold when the compound is taken with a high-fat meal (Taylor et al., 2018). CBD is highly bound to plasma proteins (>94%) and is extensively metabolized by cytochrome P450 (CYP) enzymes, mainly CYP3A4 and CYP2C19, and glucuronosyltransferases. The conversion of CBD to the primary pharmacologically active metabolite 7-hydroxy-CBD is mediated by CYP2C19, while the conversion of 7-hydroxy-CBD to the inactive 7-carboxy-CBD metabolite is mediated by CYP3A4 (Mazur et al., 2009; Jiang et al., 2011; Zendulka et al., 2016; Epidyolex, 2019; Morrison et al., 2019). CBD pharmacokinetics show prominent variability within and across patients, which is likely to contribute to large individual differences in clinical response (Franco and Perucca, 2019).

For many therapeutic agents, measurement of serum drug levels can be valuable in facilitating dose optimization. In the case of CBD, the relationship between serum levels of the parent drug and its active 7-hydroxy-metabolite and clinical effects have not been evaluated. Identification of the serum concentration at which an individual patient shows an optimal response can be used as a reference to guide dose adjustments should pharmacokinetic changes over time lead to loss of seizure control or appearance of adverse effects in a given individual (Patsalos et al., 2008). In the present article, we describe the development and validation of a novel on-line solid phase extraction high performance liquid chromatography-mass spectrometry (HPLC-MS/MS) micromethod for the simultaneous determination of CBD and 7-hydroxy-CBD in human serum and in saliva, which is suitable for pharmacokinetic studies and therapeutic drug monitoring (TDM). Although the correlation between serum and salivary CBD levels has not been investigated to date, the feasibility of using saliva samples for TDM purposes is worth exploring as most patients with Lennox-Gastaut and Dravet syndrome are children in whom avoidance of repeated venepunctures would be desirable.

## Experimental Section

### Chemicals and Reagents

**FIGURE 1:**
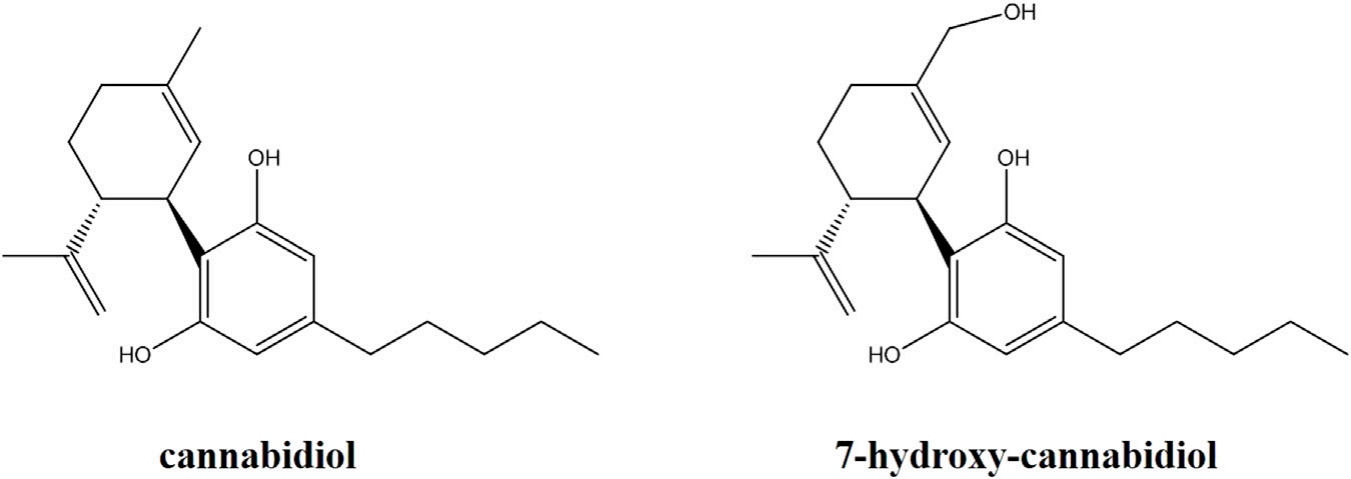
Chemical structures of cannabidiol and its active metabolite 7-hydroxy-cannabidiol.

CBD and the internal standard (IS) deuterated CBD (CBD-*d3*) were purchased from Merck (Merck, Darmstadt, Germany). 7-hydroxy-CBD was purchased from Toronto Research Chemicals (TRC, Toronto, Canada) (Figure 1). Ultrapure water for the preparation of solutions and eluents was obtained with a Millipore-Q-plus system (Millipore, Milan, Italy). LC-MS grade methanol, acetonitrile, isopropanol, 99% formic acid and 99% ammonium formate for the preparation of the mobile phase were obtained from VWR (VWR International, Milan, Italy). Drug-free human serum and saliva used for the preparation of quality control (QC) samples and calibrators were obtained from healthy adult donors and written informed consent was obtained.

### Preparation of Stock Solutions, Calibrators and QC Samples

Stock solutions of CBD (1 mg/ml), 7-hydroxy-CBD (1 mg/ml) and IS (100 μg/ml) were prepared in methanol. Working standard solutions were prepared in methanol by diluting the stock solutions to obtain concentrations of 10, 20, 50, 100, 250, 500 and 1,000 ng/ml for CBD, and 5, 10, 25, 50, 125, 250 and 500 ng/ml for 7-hydroxy-CBD. The working IS solution was prepared at 170 ng/ml. Calibrators were prepared freshly for each run by mixing 50 µl of working solutions containing both CBD and 7-hydroxy-CBD, 50 µl of working solution of IS, 50 µl of blank matrix (human serum or saliva) and 100 µl of methanol. QCs of CBD (35, 350 and 750 ng/ml) and 7-hydroxy-CBD (17.5, 175 and 375 ng/ml) were prepared from separate stock solutions and analyzed as the unknown samples.

### Sample Preparation

As for calibrators, QC samples of human serum or saliva (50 μl) were mixed with 50 μl of IS working solution (170 ng/ml), 50 μl of CBD and 7-hydroxy-CBD working solution and 100 μl of methanol. For the unknown samples, 50 μl of human serum or saliva were mixed with 50 μl of IS working solution (170 ng/ml) and 150 μl of methanol. After vortexing the mixture was centrifuged for 10 min at 4°C and 11,000 rpm using a Hettich Rotina 35R (model 1710) refrigerated centrifuge. 40 μl taken from the supernatant were injected into the HPLC-MS/MS system.

### Instrumentation and HPLC-MS/MS Parameters

The HPLC-MS/MS apparatus consisted of a 3200 QTRAP^®^ triple-quadrupole linear ion trap mass spectrometer fitted with a TurboIonSpray interface (Applied Biosystems Sciex, Darmstadt, Germany) and an HPLC ExionLC 100 integrated system equipped with a quaternary low pressure mixing pump, a column oven, an autosampler, a degasser and a controller (Applied Biosystems Sciex, Darmstadt, Germany). On-line clean-up and enrichment were performed on a perfusion column (POROS R1, 2.1 × 30 mm i.d., 20 μm, Thermo Fisher Scientific, Waltham, Massachusetts, United States). Chromatographic separation was achieved on a monolithic C18 column (Onyx, 100 × 3 mm i.d., Phenomenex, Bologna, Italy) heated at 25°C. A solution of water/methanol 98:2 v/v containing 10 mM ammonium formate and 0.1% formic acid was used as mobile phase A. Mobile phase B consisted of methanol/acetonitrile/isopropanol 80:10:10 v/v containing 8 mM ammonium formate and 0.08% formic acid. Total runtime was 12 min. The TurboIonSpray source was kept at 550°C. Electrospray ionization was performed in positive ion mode for all analytes, using the multiple reaction monitoring measurement. Setting parameters are reported in Table 1. All HPLC-MS/MS components were controlled by Analyst software version 1.6.3. MultiQuant version 3.0.2 was used for data analysis (Applied Biosystems Sciex, Darmstadt, Germany).

**TABLE 1:** Mass spectrometry parameters for CBD, 7-hydroxy-CBD and IS.

Compounds	RT (min)	Q1 mass (m/z)	Q3 mass (m/z)	DP (V)	CE (V)	CXP (V)
CBD	4.8	315.2	193.2	38	30	4
7-hydroxy-CBD	3.9	313.2	201.1	57	28	4
CBD-*d3*	4.8	318.2	196.1	38	28	4

Abbreviations: CBD, cannabidiol; 7-hydroxy-CBD, 7-hydroxy-cannabidiol; CBD-d3, cannabidiol-d3; CE, collision energy; CXP, collision cell exit potential; DP, declustering potential; IS, internal standard; RT, retention time.

### On-Line Solid Phase Extraction

On-line solid phase extraction consisted of various automated steps. In the loading step 40 µl of sample were injected by the HPLC autosampler onto the perfusion column fitted into the loading position of the 10-port switching valve. This step lasted 1 min with a flow rate of 1.5 ml/min of a mixture of 99% A: 1% B. By using this procedure the sample matrix was diverted to the waste and the analytes were retained on the perfusion column. After completing this phase and switching-back the valve, the injection step was started and the analytes were transferred to the chromatographic column. In this step the flow rate was restored at 0.5 ml/min and a gradient was set to start at 20% A: 80% B ramped to 2% A: 98% B over 3 min and then maintained for 2 min. This step permitted the separation of the analytes in the analytical column and was followed by an additional cleaning step of 3 min with 0.6 ml/min of a mixture 1% B: 99% C (mobile phase C being pure methanol) before valve activation for re-equilibration of the perfusion column at 0.6 ml/min for 3 min (99% A: 1% B).

### Method Validation

The validation of the method was performed according to the guidelines of the European Medicines Agency (EMA guidelines, 2011).

Calibration curves were obtained by evaluating the ratio between the area of the analyte peaks and the area of the IS peaks versus the corresponding concentrations of the calibrators. The curves were fitted using linear regression and the correlation coefficient was used as a measure of the goodness of fit. The concentrations of the unknown samples were calculated through the equation of the calibration line.

Precision and accuracy were investigated at four concentrations (limit of quantitation LOQ, low QC, medium QC and high QC). Precision data were expressed as coefficient of variation (CV%) with a limit of acceptability of less than 15% for QC samples and 20% for LOQ. Accuracy was calculated by comparing the means of the LOQ and QCs assay results with the nominal concentrations according to the formula [(measured value/theoretical value) x100].

Extraction recovery was determined by comparing the peak areas obtained from five different extracted QC and LOQ samples with the peak areas obtained after injection of known volumes of the non-extracted solutions containing the same concentration of CBD and 7-hydroxy-CBD. The stability of the analytes in extracted samples was evaluated by comparing the low, medium and high concentrations of fresh QC extracts with extracts at the same level of concentration stored at room temperature (24 h without protection from light), for one month at −20°C and after 3 freeze-thaw cycles.

Sensitivity was evaluated by determining the LOQ and the limit of detection (LOD). The LOQ was defined as the lowest concentration of calibrators with a signal-to-noise ratio of at least 10 with a CV% <20%. The LOD was defined as the concentration of calibrators with a signal-to-noise ratio of at least 3. Selectivity was assessed by evaluating the absence of interfering peaks at the retention time of CBD, 7-hydroxy-CBD and IS both in human serum and saliva from six different healthy individuals. Specificity was also assessed by assaying samples from individuals treated with a variety of ASMs different from CBD. Carry-over was determined by analyzing a blank solvent after the highest calibrator (six runs). Carryover was considered adequate when peak areas for CBD, 7-hydroxy-CBD and IS determined in extracted blank samples were lower than 20 and 5% than that associated with the calibration solution at the lowest concentration.

### Application to Clinical Samples

The applicability of the method was tested on samples of serum and saliva collected simultaneously from 5 patients with Lennox-Gastaut syndrome receiving different dosing regimens of a liquid formulation of pharmaceutical-grade CBD (Epidiolex^®^, 100 mg/ml CBD solution), in combination with other ASMs (Table 6). All samples were collected at steady state about 16 h after the last dose of CBD, as part of the local TDM service. Unstimulated saliva samples were aspirated with a syringe from under the tongue, transferred into 1.5 ml polypropylene tubes and stored at –20°C until analysis. Subjects rinsed their mouths with plain water and did not drink or eat for 30 min before saliva collection (Patsalos and Berry 2013).

### Statistical Analysis

Concentrations of analytes are reported as ng/ml and expressed as means ± SD. Comparison of stability parameters was performed by repeated measures analysis of variance (ANOVA). A two-tailed *p* value ≤ 0.05 was considered statistically significant. Statistical analyses were performed using SPSS version 20.0 (SPSS, Inc. Chicago, United States).

## Results

### Method Development

**FIGURE 2:**
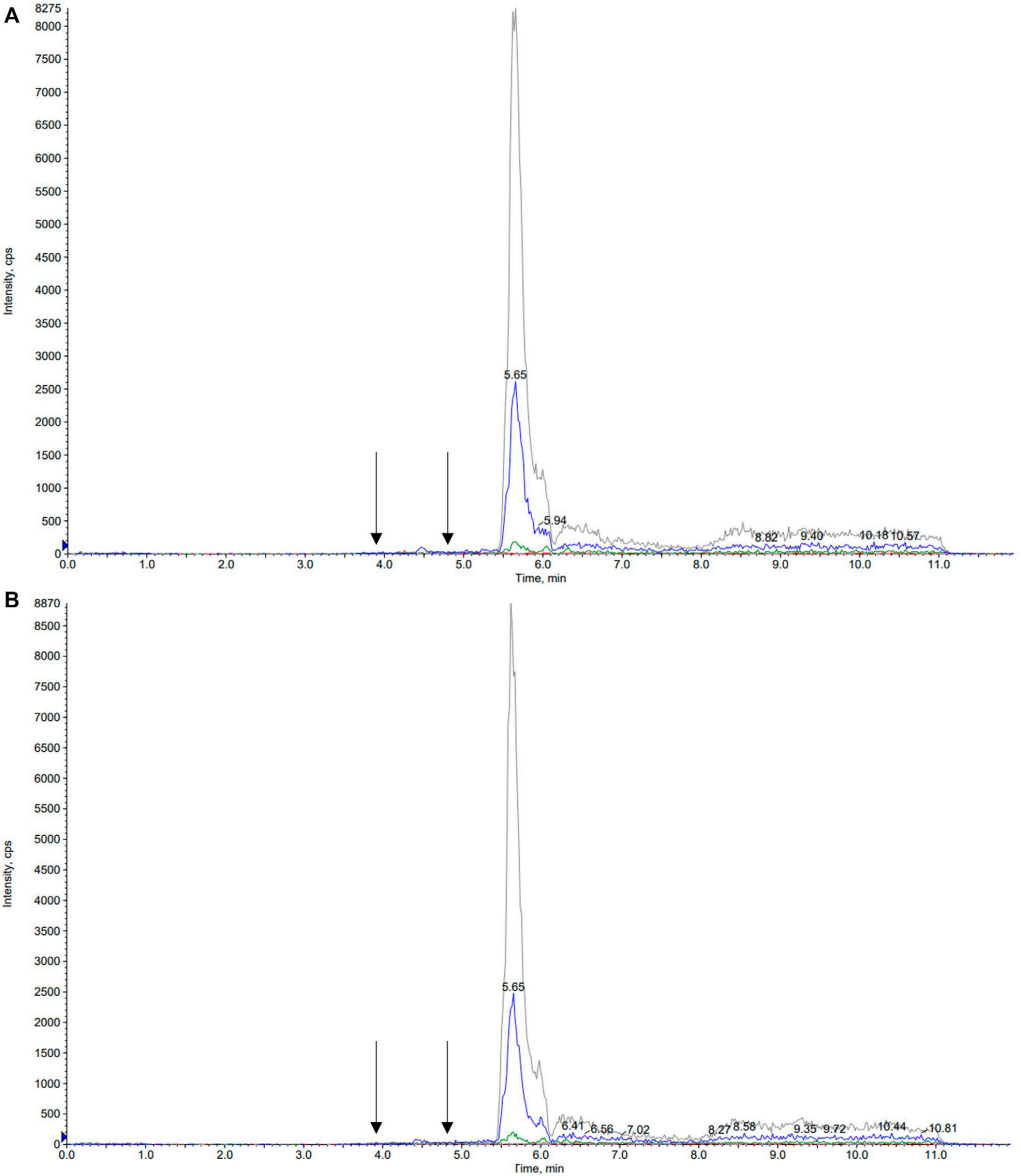
Representative chromatograms of blank serum **(A)** and saliva **(B)** samples. Arrows correspond to the retention times of 7-hydroxy-cannabidiol, cannabidiol and the internal standard cannabidiol-*d3*, respectively.

**FIGURE 3:**
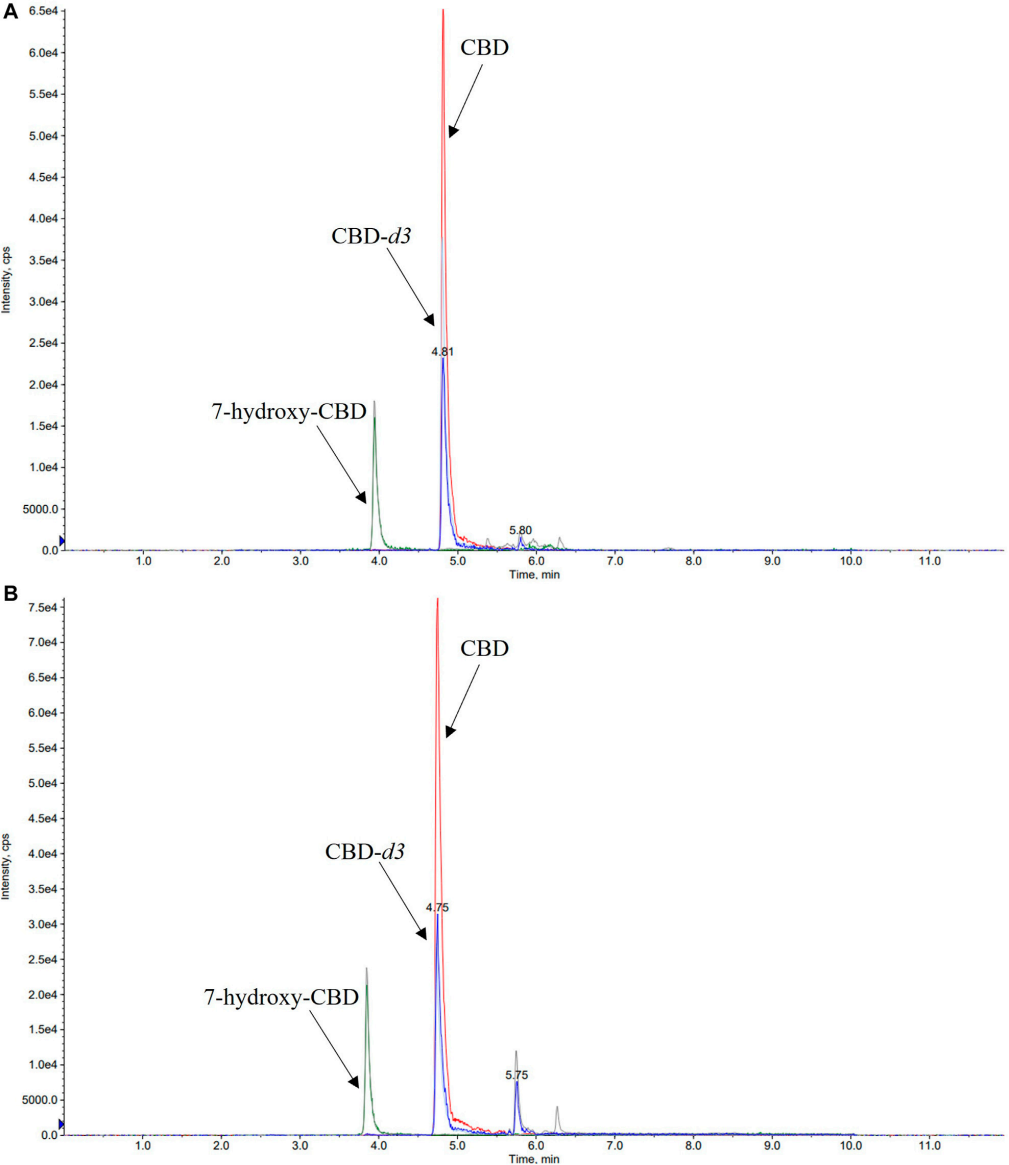
Representative chromatograms of medium QC (quality control sample spiked with CBD 350 ng/ml, 7-hydroxy-CBD 175 ng/ml and cannabidiol-*d3* 170 ng/ml) serum **(A)** and saliva **(B)** samples. CBD, cannabidiol.

**FIGURE 4:**
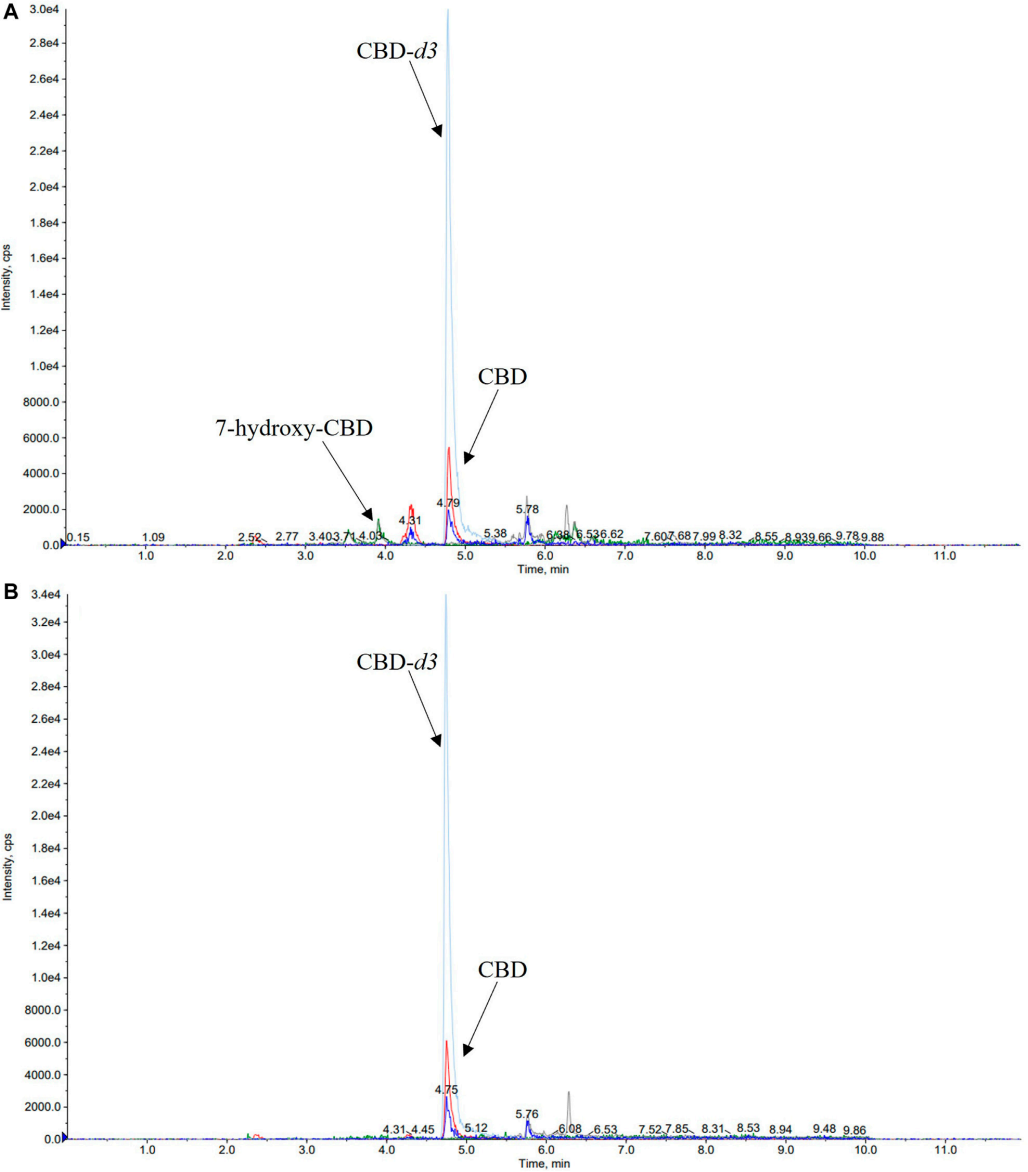
Representative chromatograms of serum **(A)** and saliva **(B)** samples of a patient taking cannabidiol (CBD) at a dosage of 6.6 mg/kg/day. The serum concentration of 7-hydroxy-CBD and CBD was 11.2 ng/ml and 22.5 ng/ml, respectively. CBD salivary concentration was 23.2 ng/ml while 7-hydroxy-CBD was not detected.

A number of different chromatographic columns were tested initially to separate the analytes, including a Zorbax (Agilent, United States) and a Kinetex column (Phenomenex, Bologna, Italy). However, only the monolithic column provided optimal peak shape and peak resolution. Under the described chromatographic conditions CBD and IS eluted at 4.8 min and 7-hydroxy-CBD eluted at 3.9 min. Representative chromatograms of extracted serum and saliva samples (blank sample, a medium QC sample and a sample from a patient receiving CBD) are shown in Figures 2–4.

### Method Validation

Within-run and between-run precision and accuracy values are reported in Tables 2 and 3. All values met the acceptability criteria specified in international guidelines for the validation of analytical assays (EMA guidelines, 2011). Intraday and interday accuracy was within 93.7–112.4% for CBD and 93.1–108.6% for 7-hydroxy-CBD in serum, and within 99.1–109.7% for CBD and 90.6–102.6% for 7-hydroxy-CBD in saliva. Intraday and interday precision values were in the range of 2.1–11.6% for CBD and 4.7–11.7% for 7-hydroxy-CBD in serum, and 1.7–6.5% for CBD and 1.4–6.8% for 7-hydroxy-CBD in saliva (Tables 2 and 3).

**TABLE 2:** Intraday and interday precision and accuracy of CBD in human serum and saliva.

Spiked concentration (ng/ml)	Measured (ng/ml)	Precision (%)	Accuracy (%)	Measured (ng/ml)	Precision (%)	Accuracy (%)
Serum	Intraday (n = 5)			Interday (n = 12)		
2.5 (LOQ)	2.5 ± 0.3	11.6	101.6	2.6 ± 0.2	7.9	104.3
35 (CQ1)	35.9 ± 2.4	6.7	102.6	32.8 ± 2.9	8.9	93.7
350 (CQ2)	380.7 ± 12.1	3.2	108.8	365.6 ± 17.5	4.8	104.5
750 (CQ3)	843 ± 17.6	2.1	112.4	794.2 ± 66.8	8.4	105.9
Saliva	Intraday (n = 5)			Interday (n = 15)		
2.5 (LOQ)	2.7 ± 0.2	6.5	106.9	2.7 ± 0.2	5.7	109.7
35 (CQ1)	37.3 ± 0.6	1.7	106.6	37.5 ± 0.9	2.5	107.1
350 (CQ2)	358.4 ± 7.1	2.0	102.4	366.4 ± 9.8	2.7	104.7
750 (CQ3)	742.9 ± 25.8	3.5	99.1	758.7 ± 24.1	3.2	101.2

**TABLE 3:** Intraday and interday precision and accuracy of 7-hydroxy-CBD in human serum and saliva.

Spiked concentration (ng/ml)	Measured (ng/ml)	Precision (%)	Accuracy (%)	Measured (ng/ml)	Precision (%)	Accuracy (%)
Serum	Intraday (n = 5)			Interday (n = 12)		
5 (LOQ)	4.7 ± 0.4	7.7	93.2	5.0 ± 0.6	11.7	100.6
17.5 (CQ1)	18.2 ± 1.6	8.9	104.1	16.3 ± 1.5	9.4	93.1
175 (CQ2)	177.1 ± 8.4	4.7	101.2	182.8 ± 12.1	6.6	104.5
375 (CQ3)	401.2 ± 30.8	7.7	107.0	407.3 ± 23.9	5.9	108.6
Saliva	Intraday (n = 5)			Interday (n = 15)		
5 (LOQ)	4.7 ± 0.3	6.6	94.7	4.5 ± 0.3	6.8	90.6
17.5 (CQ1)	17.6 ± 0.5	2.6	100.6	18.0 ± 0.7	4.1	102.6
175 (CQ2)	174.8 ± 4.3	2.5	99.9	176.0 ± 5.3	3.0	100.6
375 (CQ3)	377.4 ± 5.5	1.4	100.6	372.2 ± 11.4	3.1	99.2

Mean extraction recoveries for CBD were 81, 90, 87, 89% in serum samples and 129, 87, 100, 100% in saliva samples (LOQ, low QC, medium QC and high QC respectively). Mean extraction recoveries for LOQ, low QC, medium QC and high QC of 7-hydroxy-CBD were 113, 109, 109, 111% in serum and 108, 100, 109, 110% in saliva, respectively.

**FIGURE 5:**
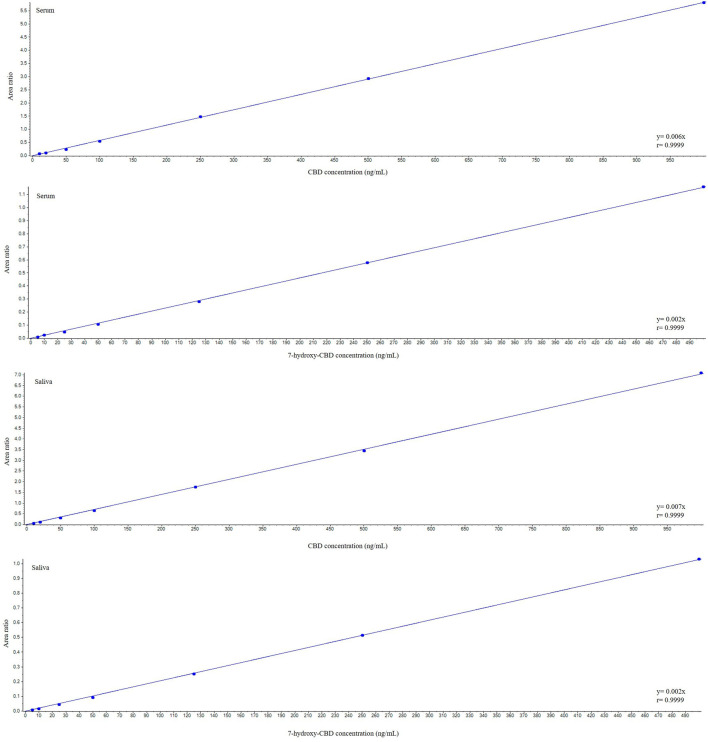
Representative calibration curves for cannabidiol and 7-hydroxy-cannabidiol. CBD, cannabidiol.

CBD and 7-hydroxy-CBD concentrations in serum and saliva were unaltered after samples storage for up to one month at −20°C and after three freeze–thaw cycles (Tables 4 and 5). Calibration curves were linear over the tested concentration range for both analytes (Figure 5). Coefficients of correlation of the curves were 0.998 and 0.999 for CBD in serum and saliva respectively, and 0.998 and 0.999 for 7-hydroxy-CBD in serum and saliva respectively. Mean slopes were 0.006 and 0.007 for CBD in serum and saliva respectively and 0.002 for 7-hydroxy-CBD in both serum and saliva. The LOQs in serum and saliva were 2.5 ng/ml for CBD and 5 ng/ml for 7-hydroxy-CBD. The LOD was 1.5 ng/ml for CBD and 7-hydroxy-CBD in both serum and saliva. No interfering peaks were detected around the retention times of CBD, 7-hydroxy-CBD and IS in serum and saliva samples. Carryover of blank injections after the highest calibrator was negligible for all the analytes.

**TABLE 4:** Stability of CBD in human serum and saliva under different storage conditions.

Spiked concentration (ng/ml)	Fresh samples (ng/ml)	24 h at room temperature (ng/ml)	Three freeze–thaw cycles (ng/ml)	1 month at −20°C (ng/ml)
Serum				
35 (CQ1)	30.9 ± 0.8	31.4 ± 0.4	32.7 ± 1.7	37.4 ± 1.6
350 (CQ2)	344.6 ± 6.7	364.9 ± 2.3	367.6 ± 7.7	364.8 ± 19.5
750 (CQ3)	706.3 ± 12.9	819.0 ± 18.1	851.3 ± 11.2	831.7 ± 30.1
Saliva				
35 (CQ1)	37.5 ± 0.6	33.7 ± 1.7	35.4 ± 1.3	39.5 ± 0.9
350 (CQ2)	355.6 ± 3.7	347.3 ± 6.6	376.5 ± 13.9	367.1 ± 18.6
750 (CQ3)	732.3 ± 11.2	748.7 ± 31.4	796.3 ± 24.1	809.1 ± 28.3

**TABLE 5:** Stability of 7-hydroxy-CBD in human serum and saliva under different storage conditions.

Spiked concentration (ng/ml)	Fresh samples (ng/ml)	24 h at room temperature (ng/ml)	Three freeze–thaw cycles (ng/ml)	1 month at −20°C (ng/ml)
Serum				
17.5 (CQ1)	15.7 ± 0.5	17.8 ± 0.9	15.2 ± 0.3	19.2 ± 1.1
175 (CQ2)	178.1 ± 14.8	191.1 ± 6.4	171.1 ± 4.3	179.6 ± 8.1
375(CQ3)	396.8 ± 11.0	415.5 ± 8.5	406.5 ± 22.3	413.0 ± 14.9
Saliva				
17.5 (CQ1)	17.6 ± 0.5	17.1 ± 0.6	17.3 ± 1.5	20.0 ± 0.2
175 (CQ2)	173.4 ± 3.5	190.6 ± 6.7	192.4 ± 5.8	192.6 ± 9.8
375 (CQ3)	376.0 ± 5.2	400.7 ± 5.9	392.7 ± 25.8	421.2 ± 10.4

### Clinical Application

Details of CBD and 7-hydroxy-CBD measurements in samples collected from 5 patients stabilized on CBD treatment are reported in Table 6. Serum CBD concentrations ranged from 22.5 to 195.5 ng/ml across patients while serum 7-hydroxy-CBD concentrations ranged from 11.2 to 80.7 ng/ml. CBD could be quantitated in all saliva samples (Figure 4). The ratio between saliva concentrations and serum concentrations was very variable (range, 9.6–103%). 7-hydroxy-CBD was below the LOQ in all saliva samples.

**TABLE 6:** Details of assay results in each of the patients receiving chronic CBD treatment. 7-hydroxy-CBD was not detected in any of the saliva samples. Abbreviations: ASMs, antiseizure medications; CBD, cannabidiol; CBZ, carbamazepine; CLB, clobazam; DLZ, delorazepam; F, female; LCM, lacosamide; LTG, lamotrigine; M, male; OXC, oxcarbazepine; PER, perampanel; PB, phenobarbital; RFN, rufinamide; TPM, topiramate; VPA, valproate.

Patient number	Age, y	Sex	Weigh, kg	Dose of CBD (mg/kg/day)	Concomitant ASMs	Serum CBD concentration (ng/ml)	Serum7-Hydroxy-CBD concentration (ng/ml)	Saliva CBD concentration (ng/ml)
1	20	F	41	15	VPA, OXC	94.4	58.5	9.1
2	18	F	52	15	PB, LTG, TPM, PER	195.5	80.7	57.8
3	18	F	57	6.6	TPM, LCM, CBZ, DLZ	22.5	11.2	23.2
4	18	M	55	8	VPA, PB, CLB	33.6	20.9	12.6
5	18	M	87	10	RFN, OXC	77.2	26.8	13.4
					VPA, CLB			

## Discussion

Several assays for determining CBD in different biological matrices such as peripheral capillary blood, plasma, serum, saliva, urine, breast milk and hair have been reported recently (Pérez Montilla et al., 2021; Sempio et al., 2021b; Cliburn et al., 2021; da Silva et al., 2020; Desrosiers et al., 2015; Cobo-Golpe et al., 2021; Reber et al., 2021). Although a few of these assays have also been validated for measuring the active metabolite 7-hydroxy-CBD in plasma or serum samples (Sempio et al., 2021a; Malaca et al., 2021), to our knowledge the present HPLC-MS/MS method is the first reported assay that permits the simultaneous determination of CBD and its active metabolite 7-hydroxy-CBD in both human serum and saliva. Performance characteristics are within acceptability standards recommended by current guidelines. The method required only 50 µl of biological fluid and retains a high sensitivity. Sample preparation involves a simple protein precipitation step followed by on-line extraction. Optimal separation of CBD and 7-hydroxy-CBD is achieved with a chromatographic run of 12 min. Use of an on-line solid phase extraction procedure associated with electrospray ionization is advantageous because it ensures optimal sensitivity/separation of the two analytes, reduces potential interferences/matrix effects and permits injection of a larger sample volume (40 µl versus standard volume of 5–10 µl) without overloading the analytical column while preserving optimal peaks shape and separation.

Our method is easy to implement in a clinical setting for several reasons. Processing and hands-on time is minimized due to absence of evaporation and/or concentration steps, without impacting on sensitivity. The assay involves direct injection of the sample in the HPLC-MS/MS system after a simple protein precipitation step with extremely low matrix effect, and automated on-line solid phase extraction avoids the time consuming process associated with off-line solid phase extraction. Stability of the analytes in samples kept at room temperature for 24 h without protection from light exposure, after three freeze/thaw cycles and after 1 month storage at −20°C facilitates the handling of clinical samples prior to the assay.

The performance characteristics of the assay will facilitate its use for TDM as well as pharmacokinetic studies, including bioavailability investigations and studies designed to assess drug interactions affecting the pharmacokinetics of CBD and its active 7-hydroxy metabolite. For many ASMs, drug concentrations in saliva are highly correlated with concentrations in serum, allowing the use of salivary samples for TDM purposes (Patsalos et al., 2008). Salivary measurements are particularly convenient for children, who often experience discomfort with repeated venepunctures. The fact that CBD is mostly used in pediatric populations stimulated us to develop a CBD assay that could also be applied to salivary samples. Our preliminary observations suggest that 7-hydroxy-CBD concentrations in saliva are very low, and that CBD saliva to serum concentrations ratios are highly variable, and may not provide a reliable estimate of the drug concentration in serum. More studies, however, are required to confirm this finding. Further studies are also required to assess the relationship between serum CBD and 7-hydroxy-CBD concentrations, antiseizure response, and adverse effects. We believe that our assay will provide a useful tool to conduct such studies.

## Conclusion

We described a novel simple, selective, sensitive and accurate on-line solid phase extraction HPLC-MS/MS method to measure CBD and 7-hydroxy-CBD in 50 µl samples of serum and saliva. The assay has been successfully validated according to existing guidelines. Fast on-line solid phase extraction on perfusion column combined with MS/MS detection enables to manage effectively background noise and matrix effect with sensitivity adequate for TDM and clinical pharmacokinetic studies.

## Data Availability

The raw data supporting the conclusion of this article can be found in Zenodo (10.5281/zenodo.6520685).
